# Smartphone-delivered multicomponent lifestyle medicine intervention for improving mental health in a nonclinical population: a randomized controlled trial

**DOI:** 10.3389/fpubh.2023.1231981

**Published:** 2024-01-16

**Authors:** Vincent Wing-Hei Wong, Jessica Tsz-Yan Tong, Nga-Kwan Shi, Chee H. Ng, Jerome Sarris, Fiona Yan-Yee Ho

**Affiliations:** ^1^Department of Psychology, The Chinese University of Hong Kong, Shatin, Hong Kong, China; ^2^Department of Psychiatry, The Melbourne Clinic and St Vincent’s Hospital, University of Melbourne, Richmond, VIC, Australia; ^3^Department of Psychiatry, Professorial Unit, The Melbourne Clinic, The University of Melbourne, Melbourne, VIC, Australia; ^4^Western Sydney University, NICM Health Research Institute, Westmead, NSW, Australia

**Keywords:** lifestyle, mood, mental health, self-help, smartphone-based intervention, randomized controlled trial

## Abstract

**Objective:**

To prevent the exacerbation of mental health burdens, a growing body of research has recommended a balanced approach that emphasizes both the delivery of mental health treatments to individuals with common mental disorders (CMDs) and the strengthening of protective factors for CMDs among nonclinical populations. This randomized controlled trial (RCT) evaluated the efficacy of a smartphone-delivered multicomponent lifestyle medicine (LM) intervention, Lifestyle Hub, for improving mental health among a nonclinical population of Chinese adults.

**Methods:**

A total of 106 participants with Patient Health Questionnaire-9 total score < 10 and Generalized Anxiety Disorder 7-Item Scale <8 were randomly assigned to either the Lifestyle Hub intervention group (LH, *n* = 53) or the waitlist control group (WL, *n* = 53). Lifestyle Hub is an 8-week smartphone-delivered multicomponent LM intervention developed based on the transtheoretical model. The intervention components included lifestyle psychoeducation, physical activity, diet and nutrition, stress management, sleep management, and motivation and goal-setting techniques. Assessments were conducted at baseline, immediate post-intervention, and 1-month follow-up (LH only).

**Results:**

The linear mixed effect model based on the intention-to-treat principle indicated that Lifestyle Hub significantly improved overall mental health, depressive symptoms, anxiety symptoms, stress, insomnia severity, overall health-promoting behaviors, dietary quality, and stress management compared to the WL group at immediate post-intervention (*d* = 0.13–0.56). No significant between-group differences were observed in terms of functional impairment, health-related quality of life, health responsibility, physical activity level, spiritual growth, and interpersonal relations. The intervention gains in the LH group were maintained at 1-month follow-up. The LH participants indicated that Lifestyle Hub was an acceptable intervention for improving mental health, although a significantly higher level of study attrition was observed in the LH group (20.8%) relative to the WL group (5.7%).

**Conclusion:**

Lifestyle Hub may serve as an efficacious and acceptable intervention for improving mental health in nonclinical adult populations. To extend the benefits of LM interventions at the population level, future studies are warranted to examine a stepped-care approach to delivering LM interventions.

**Trial registration**: This randomized controlled trial was pre-registered with ClinicalTrials.gov (NCT04295369).

## Introduction

Common mental disorders (CMDs), such as depression and anxiety, are significant public health concerns worldwide. Recent studies indicated that the prevalence of depression and anxiety among the general population ranged between 32% and 38% across the globe (1, 2). The impacts of CMDs extend beyond the individual level and can have profound adverse effects on society. It was estimated that CMDs accounted for more than 60% of disability-adjusted life-years among mental disorders and were ranked among the top 10 leading causes of years lived with disability (3). Additionally, the economic burden of CMDs is significant, with estimated costs exceeding US$1 trillion globally each year (4). The growing prevalence of CMDs reflected the inadequacy of existing strategies in addressing the ongoing mental health crisis and strengthening mental health at the population level (5). To prevent the exacerbation of mental health burdens, a growing body of research has recommended a paradigm shift in managing CMDs. Specifically, this paradigm shift advocates a balanced approach that emphasizes both the delivery of mental health treatments to individuals with CMDs and the strengthening of protective factors for CMDs among nonclinical populations (6–9).

Considering the sound evidentiary support for the relationship between lifestyles and the onset and development of CMDs, there has been a growing interest in the lifestyle medicine (LM) approach as one of the potential options for managing CMD symptoms in clinical populations and promoting mental health in nonclinical populations (10–14). The LM approach is grounded in evidence-based principles and utilizes multicomponent LM interventions to mitigate the risk of mental and physical health with a lifestyle etiology (14–17). The intervention content encompasses a range of components, including lifestyle psychoeducation and fundamental pillars such as physical activity, diet and nutrition, sleep management, stress management, and motivational elements that encourage sustained participation and engagement (12). The LM approach endeavors to empower individuals to proactively manage their own health; hence, the principal responsibility of disease management lies primarily with the individuals themselves (15).

Recent meta-analytic reviews revealed that multicomponent LM interventions comprising exercise, diet and nutrition, sleep management, and/or stress management were efficacious for improving depressive (*d* = 0.20–0.22) and anxiety symptoms (*d* = 0.19) compared to inactive control groups at immediate post-intervention (14, 16). While these meta-analyses have revealed only modest clinical effects, recent randomized controlled trials (RCTs) targeting individuals with significant depressive and anxiety symptoms have demonstrated moderate to large effect sizes for improving depressive and anxiety symptoms at immediate post-intervention assessment (*d* = 0.66–0.93) (18–21). Furthermore, multicomponent LM interventions have a robust safety profile, with recent clinical guidelines recognizing lifestyle-based interventions as a foundational component for the prevention, treatment, and recovery of CMDs (11, 22–24). Although the efficacy of multicomponent LM interventions for improving CMD symptoms in the nonclinical population has been demonstrated, such interventions were predominantly delivered face-to-face by health professionals, which restricted access in larger population groups (11, 14, 16, 17). Therefore, novel and scalable modes of delivery are required to meet the vast mental health needs of the population.

The utilization of smartphones has emerged as a promising approach for augmenting the dissemination and reach of LM interventions. With an estimated 80% of the world population being smartphone users (13), this ubiquitous technology represents an accessible medium to facilitate population-level mental health promotion. Additionally, smartphone-delivered interventions can overcome geographical and time constraints and are more affordable compared to face-to-face interventions (25). Furthermore, smartphone-delivered interventions can provide a level of anonymity and privacy, which is crucial for individuals who are concerned with mental health-related stigma (26). Sound evidence from meta-analyses suggested that smartphone-delivered mental health interventions were promising for improving depressive symptoms (*g* = 0.24), anxiety symptoms (*g* = 0.24–0.28), and stress (*g* = 0.36) relative to active and inactive control groups among nonclinical populations at immediate post-intervention assessment (27, 28).

Given the potential merit of smartphone-delivered interventions in managing CMDs, a pioneering smartphone-delivered LM intervention, Lifestyle Hub, was developed (21). The efficacy of Lifestyle Hub in ameliorating depressive symptoms has previously been evaluated in a Chinese adult population with at least moderate levels of depressive symptoms (21). The results suggested that Lifestyle Hub was efficacious in improving depressive and anxiety symptoms with moderate to large effect sizes compared to a waitlist (WL) control group at immediate post-intervention assessment (*d* = 0.66–0.93). Moreover, participants who used the intervention for 8 weeks generally considered Lifestyle Hub as acceptable and creditable for improving depressive symptoms. Building upon the positive findings and recognizing the dearth of literature on the efficacy of smartphone-delivered multicomponent LM for improving mental health among nonclinical populations, we conducted the first RCT to evaluate a smartphone-delivered, 8-week multicomponent LM intervention, Lifestyle Hub, for improving mental health among a Chinese nonclinical population. We hypothesized that Lifestyle Hub would result in significant improvements in overall mental health conditions (including depressive symptoms, anxiety symptoms, stress, and perceived insomnia severity), functional impairment, health-related quality of life (HRQOL), and overall health-promoting behaviors (HPBs) relative to a WL control group at immediate post-intervention (Week 9). In addition, we hypothesized that these intervention gains would be maintained at the 1-month follow-up assessment (Week 13).

## Methods

### Study design

To assess the efficacy of Lifestyle Hub in improving mental health, a two-arm RCT was conducted between February and May 2020. A total of 106 eligible participants were randomly assigned to either the intervention group receiving the 8-week smartphone-delivered multicomponent LM intervention (Lifestyle Hub; LH) or the WL control group. This study was approved by the Survey and Behavioral Research Ethics Committee (Reference no. SBRE-19-303), The Chinese University of Hong Kong. The trial was registered with ClinicalTrials.gov (NCT04295369).

### Eligibility criteria

Participants were eligible if they (1) were Hong Kong residents; (2) aged 18 years or older; (3) were able to read Chinese and type in Chinese or English; (4) had an internet-enabled mobile device (iOS or Android operating system); and (5) were willing to provide informed consent and comply with the trial protocol. Participants were excluded if they (1) had a Patient Health Questionnaire-9 (PHQ-9) total score ≥ 10, indicating the presence of at least a moderate level of depressive symptoms (29, 30); (2) had a Generalized Anxiety Disorder 7-Item Scale (GAD-7) total score ≥ 8, indicating the presence of at least a mild level of anxiety symptoms (29, 31); (3) were receiving psychotherapy and/or unstable medication for depression and/or anxiety disorders in the past 2 months; (4) had a PHQ-9 Item 9 score > 2, indicating current suicidality that might require active crisis management (24-h suicide prevention hotlines and details of accessing public professional mental health services were offered); (5) had an unstable medical condition or were not recommended for lifestyle modifications by health professionals (e.g., physician, dietitian); or (6) were having major psychiatric, medical or neurocognitive disorders that made intervention involvement difficult or might interfere with participation in the intervention or adherence to lifestyle modification.

### Recruitment and study procedure

Participants were recruited via the university mass mailing system, social networking websites (i.e., Facebook and Instagram), and print media. Prospective participants were required to complete a set of online questionnaires for screening purposes, which included (1) the PHQ-9 measuring depressive symptoms and current suicidality; (2) the GAD-7 measuring anxiety symptoms, (3) a self-report checklist on eligibility criteria, and (4) a demographics questionnaire. Eligible participants were invited to participate in this study via text messaging or telephone calls by a research assistant. Besides, they were instructed to download an in-house smartphone application (*Longitudinax*) for online informed consent and data collection. Participants who signed the online consent form and completed the baseline questionnaire were randomly assigned to either the LH or WL group in a 1:1 ratio by an independent statistician using a computer-generated list of numbers.

Given the nature of the study design, blinding of participants and research personnel was not possible. However, the data analyst was blinded to the group assignment. The research assistant instructed the participants in the LH group to download Lifestyle Hub, and each LH participant was provided with a unique account via text messages. The LH participants were informed that Lifestyle Hub is a self-help intervention such that no therapeutic support would be provided throughout the trial period. However, they could contact the research assistant for technical assistance (e.g., log-in problems). Participants assigned to the WL group were informed that they would be given access to Lifestyle Hub upon the completion of the immediate post-intervention assessment at Week 9. A research compensation of HK$100 (approximately USD12.8) was offered to the participants in both groups after they completed all the required assessments.

### The LH intervention group

The detailed intervention content has been published elsewhere (21) and summarized in Table 1. Lifestyle Hub is an 8-week smartphone-delivered multicomponent LM intervention developed based on the transtheoretical model (32). The intervention components included (1) lifestyle psychoeducation; (2) physical activity; (3) nutrition; (4) stress management; (5) sleep management; and (6) motivation and goal-setting techniques. Specifically, Sessions 1 and 2 were designed to enhance participants’ perception of their current lifestyle and raise doubts about problematic lifestyle behavior(s). Sessions 3 and 4 aimed to facilitate the identification of the pros and cons of unhealthy lifestyle habits and the development of personal motivations for lifestyle modifications. Sessions 5 and 6 focused on preparing and establishing a practical action plan for lifestyle modifications. Session 7 aims to strengthen participants’ self-efficacy in overcoming obstacles and reaffirm the long-term benefits of lifestyle modifications. The last session was intended to consolidate the implemented lifestyle modifications and prevent relapse in the long term.

**Table 1:** Overview of the intervention structure and content of Lifestyle Hub.

Session	Content	Homework activity
1–2	Overview of *Lifestyle Hub* Introduction to lifestyle medicine A brief assessment of physical activity Introduction to low-intensity exercise with demonstration videos Explain the association between physical activity and mental health Introduction to calories (with gamified tests) Tips for healthy eating Explain the relationship between food micronutrients and mental health SMART goal-setting	Setting up mid-term and short-term goals Daily lifestyle tasks (physical activity and diet)
3–4	Introduction to low-intensity exercise with demonstration videos (i.e., flexibility and balancing exercise) Introduction to food nutrition labels Introduction to progressive muscle relaxation Explain the association between sleep and mental health	Setting up short-term goals Daily lifestyle tasks (physical activity and diet) Progressive muscle relaxation
5–6	Introduction to moderate-intensity exercise with demonstration videos (i.e., cardiovascular and muscle training) Wake-up and wind-down routine Sleep hygiene and sleep–wake regularity Stimulus control Worry time Problem-solving strategies	Setting up short-term goals Daily lifestyle tasks (physical activity and diet) Wake-up and wind-down routine practice Worry time and problem-solving practice
7	Introduction to yoga and abdominal breathing exercise Explain the association between mindfulness and mental health Introduction to positive psychology	Setting up short-term goals Daily lifestyle tasks (physical activity and diet) Mindfulness and abdominal breathing practice Gratitude journal
8	Revision of all session content Review of *Lifestyle Hub* Review self-setting goals and lifestyle modification progress Setting long-term goals	Setting up long-term goals Daily practice of lifestyle modifications

To facilitate intervention delivery and participant understanding, the eight 60-min weekly sessions were divided into 45 submodules (i.e., 5–6 submodules per session), and the content was structured to progress from low to high intensity. Each weekly session began with a review of the previous session to consolidate participant learning outcomes (except for Session 1). Subsequently, new intervention content was introduced through animated videos (8–15 min each with video scripts supplemented), gamified mini quizzes, texts, audios, and/or infographics. Each weekly session was concluded with a smart goal-setting submodule to facilitate short/long-term lifestyle modifications and self-monitoring of intervention progress. The motivational interviewing approach was adopted to promote lifestyle modifications and guide participants to accomplish their lifestyle goals (33). To encourage self-monitoring of lifestyle behaviors and facilitate long-term lifestyle modifications, daily homework activities (10–20 min per day) were pre-assigned, and a daily challenge (e.g., walking 8,000 steps a day) was automatically sent to participants every morning to promote adherence to the intervention. Besides, a wide variety of extra materials regarding exercise (e.g., low-intensity exercise demonstrations and yoga), diet (recipes with cooking demonstrations), sleep management, and stress management (e.g., video demonstrations of progressive muscle relaxation and diaphragmatic breathing) were continuously provided in the Explore page of Lifestyle Hub. Moreover, participants were able to set personalized short-term and long-term lifestyle goals using the “Goal Setting” function.

### The WL control group

Participants allocated to the WL control group were advised to maintain their usual lifestyle routines and were given access to Lifestyle Hub upon the completion of the immediate post-intervention assessment at Week 9.

### Outcome measures

Self-report outcome measures were collected at baseline (Week 0), immediate post-intervention (Week 9), and 1-month post-intervention (LH only; Week 13). The primary outcome was overall mental health conditions as assessed by the Chinese version of Depression Anxiety Stress Scales-21 (DASS-21) (29, 34, 35). DASS-21 is a 21-item self-report questionnaire assessing overall mental health conditions over the past week on a 4-point Likert scale. The possible responses ranged from “0” (did not apply to me at all) to “3” (applied to me most of the time). The overall mental health condition score was the sum of the 21 items (range = 0–63). The raw score obtained was multiplied by 2 to compute the final score. The lower the score, the better the overall mental health conditions. DASS-21 has shown good internal consistency for the depression (Cronbach’s alpha = 0.83), anxiety (Cronbach’s alpha = 0.80), and stress (Cronbach’s alpha = 0.82) subscales (35).

The secondary outcomes included depressive symptoms, anxiety symptoms, stress levels, insomnia severity, functional disability, HRQOL, HPBs, and intervention acceptability. The Chinese version of the Insomnia Severity Index (ISI) (36) was employed to measure perceived insomnia severity and the associated impairment on a 5-point Likert scale, ranging from “0” (no problem) to “4” (very severe problem). The total score (range = 0–28) was calculated by summing up the 7-item scores. The higher the sum, the higher the perceived severity of insomnia symptoms. The Chinese version of ISI has demonstrated satisfactory psychometric properties (Cronbach’s alpha = 0.83) (37).

Functional disability was measured by the Chinese version of the Sheehan Disability Scale (SDS) on an 11-point Likert scale (38). SDS is a 3-item scale that assesses functional impairment in three domains: work or school, social life, and family life (38). The sum of the 3-item scores represents a single-dimensional measure of global functional impairment that ranges from 0 (unimpaired) to 30 (highly impaired). The Chinese version of the SDS has demonstrated good psychometric properties (Cronbach’s alpha = 0.89) (39).

HRQOL was measured by the Hong Kong version of the Short Form (Six-Dimension) Health Survey (SF-6D) (40). SF-6D is a preference-based single index measure of health in six dimensions, encompassing physical functioning, role limitation, social functioning, bodily pain, mental health, and vitality. The total score was calculated using a scoring algorithm based on the Hong Kong population norms, with a range of 0.315 (the worst HRQOL) to 1 (full health) (41). The Hong Kong version of SF-6D has demonstrated adequate psychometric properties (Cronbach’s alpha = 0.71) (40).

HPBs were assessed using the Chinese version of the Health-Promoting Lifestyle Profile (HPLP-II) (42). HPLP-II is a 52-item scale designed to evaluate overall health-promoting lifestyle and six specific domains of HPBs, including spiritual growth, interpersonal relations, nutrition, physical activity, health responsibility, and stress management, on a 4-point Likert scale ranging from 1 (never) to 4 (routinely). The sum of the scores on the 52 items yields the overall health-promoting lifestyle score (range = 52–208), whereas the specific domain scores were computed by adding the scores of respective items (range = 8–32 or 9–36). The higher the score, the more the HPBs. The Chinese version of HPLP-II has demonstrated very good psychometric properties in an adult Chinese Hong Kong population (Cronbach’s alpha = 0.95) (21).

The Chinese version of the Credibility-Expectancy Questionnaire (CEQ) was adopted to evaluate intervention acceptability (43). CEQ is a 6-item scale, in which the mean of the first three items yielded a rating of intervention credibility, whereas the mean of the remaining three items was for intervention expectancy. The higher the scores, the higher the intervention credibility and success expectancy. The Chinese version of the CEQ has demonstrated adequate psychometric properties in an adult Chinese Hong Kong population (Cronbach’s alpha = 0.74–0.80) (21).

### Statistical analysis

Sample size estimation was conducted using G*Power 3 (44). Based on a 5% α error probability and 80% power in a two-tailed test and accounting for an anticipated study attrition rate of 20% (14, 16), an estimated total sample size of 96 (i.e., 48 in each group) was considered necessary to detect a between-group difference of 0.66 in depression and anxiety symptoms as measured by DASS-21 (18, 21).

The R version 4.1.2 (45) was used to conduct statistical analyses. Statistical significance was determined using two-tailed tests with a value of *p* of less than 0.05. Cohen’s *d* was used as the effect size measure, with magnitudes of 0.2 considered as a small effect, 0.5 as a medium effect, and 0.8 as a large effect (46). Between-group differences of baseline characteristics were assessed using independent-samples *t*-test or chi-square test of independence. The efficacy of Lifestyle Hub on various outcome measures (except intervention acceptability) from baseline (Week 0) to immediate post-intervention (Week 9) was evaluated using a linear mixed-effects model (LMM) based on the intention-to-treat principle. The LMM employs maximum likelihood estimation and assumes data is missing at random (47, 48). The durability of Lifestyle Hub from immediate post-intervention (Week 9) to 1-month follow-up assessments (Week 13) as well as intervention acceptability in the LH were assessed using paired-samples *t*-tests. Study attrition was defined as the number of dropouts throughout the entire study, which comprised dropouts during the intervention, at immediate post-intervention (Week 9), and at 1-month follow-up (Week 13) assessments. The between-group difference in study attrition was estimated using the chi-square test of independence. Intervention usage was defined as the amount of time participants spent on the eight 60-min weekly sessions. Specifically, participants were considered to have completed a session if a minimum of 60 min were spent. In addition, submodule completion was reported in mean and cumulative percentages.

## Results

### Participant characteristics

**Figure 1:**
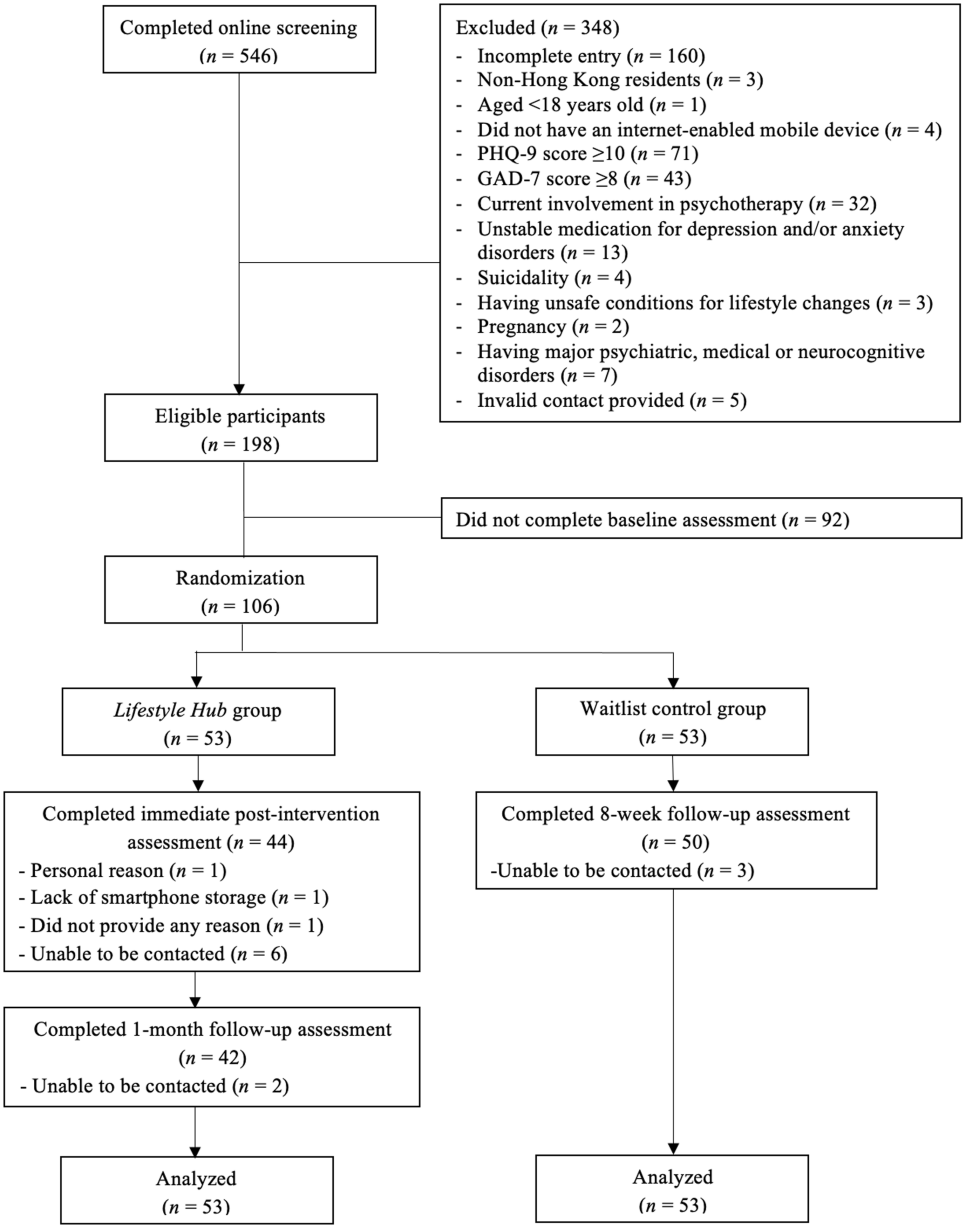
CONSORT flow diagram.

In sum, 546 prospective participants completed the online screening for eligibility, of which 348 were excluded due to a variety of reasons (Figure 1). The 198 eligible individuals were invited to complete the baseline assessment. Among them, 106 individuals completed the baseline assessment and were randomly assigned to either the LH intervention group (*n* = 53) or the WL control group (*n* = 53). The mean age of the participants was 35.7 years (*SD* = 12.0), and the majority of participants were female (77.4%). The participants, in general, had a normal level of DASS-21 measured depressive (mean = 7.2; *SD* = 6.4) and anxiety (mean = 6.6; *SD* = 5.6) symptoms as well as a moderate level of stress (mean = 11.8; *SD* = 6.7) at baseline. There was no significant difference between the LH and the WL groups in any baseline characteristics (*p*s > 0.05; Table 2).

**Table 2:** Baseline characteristics.

Variable	LH (*n* = 53)	WL (*n* = 53)	Total (*n* = 106)	*p*-value
Age, years	34.36 (12.20)	37.02 (11.72)	35.69 (11.98)	0.26
Female, *n* (%)	41 (77.36)	41 (77.36)	82 (77.36)	1
Level of education *n*, (%)				0.62
Primary or below	0 (0)	0 (0)	0 (0)	
Junior secondary	0 (0)	1 (1.89)	1 (0.94)	
Senior secondary	3 (5.66)	7 (13.21)	10 (9.43)	
Diploma/certificate	5 (9.43)	3 (5.66)	8 (7.55)	
Associate degree	3 (5.66)	4 (7.55)	7 (6.60)	
Bachelor’s degree	23 (43.40)	22 (41.51)	45 (42.45)	
Master’s degree or above	19 (35.85)	16 (30.19)	35 (33.02)	
Marital Status *n*, (%)				0.42
Single	38 (71.70)	33 (62.26)	71 (66.98)	
Married	12 (22.64)	18 (33.96)	30 (28.30)	
Divorced/widowed	3 (5.66)	2 (3.77)	5 (4.72)	
Number of children *n*, (%)				0.57
0	42 (79.25)	39 (73.58)	81 (76.42)	
1	6 (11.32)	7 (13.21)	13 (12.26)	
≥2	5 (9.43)	7 (13.21)	12 (11.32)	
Employment status *n*, (%)				0.23
Full-time	28 (52.83)	36 (67.92)	64 (60.38)	
Part-time	12 (22.64)	10 (18.87)	22 (20.75)	
Not applicable	13 (24.53)	7 (13.21)	20 (18.87)	
Monthly income *n*, (%)				0.05
≤HK$ 5,000	21 (39.62)	11 (20.75)	32 (30.19)	
HK$ 5,001 - 10,000	3 (5.66)	4 (7.55)	7 (6.60)	
HK$ 10,001 - 20,000	8 (15.09)	10 (18.87)	18 (16.98)	
HK$ 20,001 - 30,000	3 (5.66)	13 (24.53)	16 (15.09)	
HK$ 30,001 - 50,000	12 (22.64)	13 (24.53)	25 (23.58)	
HK$ 50,001 - 70,000	4 (7.55)	2 (3.77)	6 (5.66)	
HK$ 70,001 - 90,000	0 (0)	0 (0)	0 (0)	
> HK$ 90,000	2 (3.77)	0 (0)	2 (1.89)	
DASS-21				
Total	25.02 (15.78)	26.30 (15.81)	25.66 (15.73)	0.68
Anxiety	6.94 (6.12)	6.30 (5.10)	6.62 (5.62)	0.56
Depression	6.53 (6.23)	7.92 (6.53)	7.23 (6.38)	0.26
Stress	11.56 (6.96)	12.08 (6.50)	11.81 (6.71)	0.69
CEQ				
Credibility	5.73 (1.28)	5.82 (1.57)	5.78 (1.43)	0.74
Expectancy (%)	49.09 (16.88)	51.55 (19.23)	50.32 (18.04)	0.49
SDS	2.40 (3.24)	3.72 (4.83)	3.06 (4.14)	0.10
ISI	6.96 (4.13)	6.30 (4.68)	6.63 (4.41)	0.44
SF-6D	0.79 (0.11)	0.76 (0.13)	0.78 (0.12)	0.24
HPLP-II				
Total	67.49 (17.07)	66.06 (22.43)	66.77 (19.38)	0.72
Health Responsibility	8.32 (3.78)	8.06 (4.88)	8.19 (4.34)	0.76
Physical Activity	8.98 (4.02)	7.96 (4.84)	8.47 (4.45)	0.24
Nutrition	12.62 (3.65)	12.98 (4.37)	12.80 (4.01)	0.65
Spiritual Growth	13.58 (4.91)	13.13 (5.27)	13.36 (5.07)	0.65
Interpersonal Relations	13.79 (4.70)	13.43 (4.33)	13.61 (4.50)	0.68
Stress Management	10.19 (3.22)	10.49 (4.22)	10.34 (3.74)	0.68

Data were presented as mean (*SD*) unless otherwise specified. CEQ, Credibility-Expectancy Questionnaire; DASS-21, Depression Anxiety Stress Scales-21; HPLP-II, Health-Promoting Lifestyle Profile; ISI, Insomnia Severity Index; LH, Lifestyle medicine intervention group; SDS, Sheehan Disability Scale; SF-6D, Short Form (Six-Dimension) Health Survey; WL, Waitlist control group.

### Intervention dropout

The study attrition rates (i.e., the number of dropouts throughout the entire study period) of the LH and WL groups were 20.8% (*n* = 11) and 5.7% (*n* = 3), respectively. The chi-square test of independence suggested there were statistically significant differences in study attrition between the two groups (χ^2^ = 4.03, *p* < 0.05). Specifically, 3 LH participants withdrew during the eight-week intervention period. The reasons for withdrawal included personal reasons (*n* = 1) and lack of smartphone storage (*n* = 1), while the remaining participant did not provide any reason. At the immediate post-intervention and 1-month follow-up, an additional 8 LH participants withdrew from this study because they were uncontactable. For the WL group, 3 participants could not be reached at immediate post-intervention.

### Intervention usage

At Week 9, the 44 LH participants who completed the immediate post-intervention assessment had a mean Lifestyle Hub utilization of 11 days (*SD* = 8.2) and a mean submodule completion rate of 57.8% (i.e., 26 out of 45 submodules, *SD* = 15.5). Furthermore, the submodule completion was measured cumulatively. Specifically, 4 participants (9.1%) had completed all submodules, 25 participants (56.8%) completed at least 70% of submodules, 28 participants (63.6%) completed at least 50% of submodules, and 35 participants (79.5%) completed at least 30% of submodules.

### Between-group comparisons

The LMM analyses revealed that participants using the Lifestyle Hub had significant improvement in overall mental health conditions (*d* = 0.52, *p* < 0.01), depressive symptoms (*d* = 0.56, *p* < 0.05), anxiety symptoms (*d* = 0.33, *p* < 0.01), stress (*d* = 0.44, *p* < 0.05), insomnia severity (*d* = 0.29, *p* < 0.01), overall HPBs (*d* = 0.31, *p* < 0.01), dietary quality (*d* = 0.13, *p* < 0.05), and stress management (*d* = 0.36, *p* < 0.001) from baseline to immediate post-intervention relative to the WL control group. However, no significant between-group difference was observed in functional impairment, HRQOL, health responsibility, physical activity level, spiritual growth, and interpersonal relations (*p*s > 0.05; Table 3).

**Table 3:** Effects of Lifestyle Hub at the immediate post-intervention assessment (Week 9) (based on the intention-to-treat principle).

Outcomes	Lifestyle Hub group			Waitlist control group			*p*-value	Between-group effect size
	Baseline *M (SD)*^a^	Post-intervention *M (SD)*^b^	Within-group effect size	Baseline *M (SD)*^a^	Post-intervention *M (SD)*^c^	Within-group effect size	Group x time effect	
DASS-21								
Total Score	25.02 (15.78)	19.59 (13.17)	0.41	26.30 (15.81)	28.12 (18.90)	0.06	<0.01**	0.52
Anxiety	6.94 (6.12)	5.23 (4.47)	0.32	6.30 (5.10)	6.96 (5.75)	0.08	<0.01**	0.33
Depression	6.53 (6.23)	4.82 (4.69)	0.32	7.92 (6.53)	8.64 (8.24)	0.05	<0.05*	0.56
Stress	11.56 (6.96)	9.55 (6.64)	0.37	12.08 (6.50)	12.52 (6.98)	0.04	<0.05*	0.44
CEQ								
Credibility	5.73 (1.28)	6.58 (1.34)	0.60	5.82 (1.57)	–	–	–	–
Expectancy (%)	49.09 (16.88)	54.63 (16.85)	0.22	51.55 (19.23)	–	–	–	–
SDS	2.40 (3.24)	2.77 (4.80)	0.18	3.72 (4.83)	4.62 (5.91)	0.15	0.73	0.34
ISI	6.96 (4.13)	6.16 (4.98)	0.11	6.30 (4.68)	7.68 (5.60)	0.28	<0.01**	0.29
SF-6D	0.79 (0.11)	0.82 (0.09)	0.21	0.76 (0.13)	0.76 (0.13)	0.01	0.22	0.49
HPLP-II								
Total	67.49 (17.07)	73.07 (16.36)	0.45	66.06 (22.43)	66.34 (25.69)	0.00	<0.01**	0.31
Health Responsibility	8.32 (3.78)	8.66 (3.65)	0.16	8.06 (4.88)	8.30 (5.39)	0.04	0.61	0.08
Physical Activity	8.98 (4.02)	9.91 (3.39)	0.37	7.96 (4.84)	7.94 (5.22)	0.00	0.06	0.44
Nutrition	12.62 (3.65)	13.98 (3.39)	0.48	12.98 (4.37)	13.42 (4.86)	0.07	<0.05*	0.13
Spiritual Growth	13.58 (4.91)	14.05 (4.32)	0.22	13.13 (5.27)	12.90 (5.82)	0.03	0.08	0.22
Interpersonal Relations	13.79 (4.70)	14.41 (4.26)	0.16	13.43 (4.33)	13.16 (4.72)	0.08	0.10	0.28
Stress Management	10.19 (3.22)	12.07 (3.53)	0.64	10.49 (4.22)	10.62 (4.42)	0.02	< 0.001***	0.36

DASS-21, Depression Anxiety Stress Scales-21; CEQ, Credibility-Expectancy Questionnaire; SDS, Sheehan Disability Scale; ISI, Insomnia Severity Index; SF-6D, Short Form (Six-Dimension) Health Survey; HPLP-II, Health-Promoting Lifestyle Profile. *p < 0.05; **p < 0.01, ***p < 0.001.

a *n* = 53.

b *n* = 44.

c *n* = 50.

### Within-group comparisons

The paired-samples *t*-tests showed that there were no significant differences in any outcomes from immediate post-intervention (Week 9) to 1-month follow-up (Week 13) in the LH group (*p*s > 0.05; Table 4).

**Table 4:** Effects of Lifestyle Hub at the 1-month follow-up assessment (Week 13).

	Post-intervention *M (SD)*^a^	Follow-up *M (SD)*^b^	Within-group effect size (*d*)	*p*-value
DASS-21				
Total Score	19.59 (13.17)	20.05 (15.40)	0.06	0.67
Anxiety	5.23 (4.47)	5.43 (5.00)	0.14	0.36
Depression	4.82 (4.69)	5.14 (5.66)	0.05	0.73
Stress	9.55 (6.64)	9.48 (6.52)	0.01	0.96
SDS	2.77 (4.80)	2.76 (4.37)	0.00	1
ISI	6.16 (4.98)	6.07 (4.40)	0.01	0.96
SF-6D	0.82 (0.09)	0.81 (0.13)	0.07	0.70
HPLP-II				
Total	73.07 (16.36)	74.02 (20.08)	0.06	0.58
Health Responsibility	8.66 (3.65)	9.81 (4.92)	0.25	0.06
Physical Activity	9.91 (3.39)	10.31 (3.29)	0.14	0.32
Nutrition	13.98 (3.39)	14.29 (3.95)	0.06	0.63
Spiritual Growth	14.05 (4.32)	13.76 (4.99)	0.01	0.96
Interpersonal Relations	14.41 (4.26)	14.24 (4.83)	0.05	0.64
Stress Management	12.07 (3.53)	11.62 (3.22)	0.12	0.27

DASS-21, Depression Anxiety Stress Scales-21; SDS, Sheehan Disability Scale; ISI, Insomnia Severity Index; SF-6D, Short Form (Six-Dimension) Health Survey; HPLP-II, Health-Promoting Lifestyle Profile.

a *n* = 44.

b *n* = 42.

### Intervention acceptability

The paired-samples *t*-test revealed a significant within-group difference in intervention credibility [*t*(43) = −3.12, *p* < 0.01] from baseline to immediate post-intervention in the LH (Week 9). No significant difference in intervention expectancy was observed [*t*(43) = −1.24, *p* = 0.22; Table 3].

## Discussion

This RCT examined the efficacy and acceptability of a smartphone-delivered multicomponent LM intervention, Lifestyle Hub, for improving mental health among a nonclinical population of Chinese adults. The results indicated that Lifestyle Hub had small to moderate effects (*d* = 0.29–0.56) in improving overall mental health conditions, depressive symptoms, anxiety symptoms, stress, and perceived insomnia severity relative to the WL control group at immediate post-intervention. In addition, the LH group had significant improvement in overall HPBs, dietary quality, and stress management compared with the WL control group at immediate post-intervention (*d* = 0.13–0.36). Contrary to our hypotheses, no significant between-group differences were observed in functional impairment, HRQOL, health responsibility, physical activity level, spiritual growth, and interpersonal relations. The intervention gains in the LH group were maintained at the 1-month follow-up. Overall, the LH participants indicated that Lifestyle Hub was an acceptable intervention for improving mental health, although a statistically significantly higher level of study attrition was observed in the LH group relative to the WL group.

The findings regarding the improvement in depressive symptoms (*d* = 0.56), anxiety symptoms (*d* = 0.33), and perceived insomnia severity (*d* = 0.29) at immediate post-intervention are in line with our previous RCT examining the efficacy of Lifestyle Hub in managing depressive symptoms among Chinese adults with at least moderate depressive symptomatology (21). Notably, these effect sizes are generally superior to previous meta-analyses examining the effects of multicomponent LM intervention on improving depressive symptoms (*d* = 0.16), anxiety symptoms (*d* = 0.14), and insomnia symptoms (*d* = 0.32) in diverse populations (14, 16, 17). Overall, our findings suggest that Lifestyle Hub can simultaneously improve CMD symptoms, stress, and perceived insomnia severity, even within a nonclinical sample where perhaps limited room for improvement is anticipated (14, 16, 17). This encouraging evidence provided preliminary support for the proposition that multicomponent LM interventions may serve as a transdiagnostic health management intervention for CMDs (17, 49, 50). However, more studies are needed to draw a definitive conclusion regarding the transdiagnosticity of the LM approach across these disorders (17).

Contrary to the hypothesis, Lifestyle Hub did not result in a significant improvement in health responsibility compared to the WL control group at immediate post-intervention. This finding is noteworthy, as our previous RCT found a moderate to large between-group improvement in health responsibility at immediate post-intervention (*d* = 0.78) (21). Previous studies suggested that concerns about health responsibility tend to emerge when individuals become ill (e.g., diagnosed with a health problem, experienced active symptoms, or received feedback from a health professional) or are able to acknowledge the potential link between their past behaviors and current health issues (51, 52). Therefore, a speculative reason for the contrasting results is that the nonclinical population in this RCT was less likely to connect their current lifestyle choices with their mental health conditions, resulting in a lower level of health responsibility compared to our previous RCT which targeted depressed individuals. Promoting health responsibility is important within the LM approach. A higher level of health responsibility could empower individuals to engage in more HPBs (53), thereby maximizing intervention outcomes and sustaining intervention gains in the long run (15). To improve health responsibility among nonclinical populations, future smartphone-delivered multicomponent LM interventions could incorporate more comprehensive lifestyle psychoeducation regarding the relationship between lifestyle choices and the development of CMDs. Moreover, structured self-reflection questions could be utilized to raise their awareness and strengthen their motivation to engage in healthy lifestyles (54).

Furthermore, our results demonstrated nonsignificant group-by-time interaction in functional impairment, HRQOL, spiritual growth, and physical activity level between the LH and WL groups, while the previous RCT found significant improvements in these outcomes with small to large effect sizes (*d* = 0.11–0.89) (21). The observed discrepancies may be attributed to floor effects, given the current study had comparatively low scores in SDS and high scores in HRQOL, spiritual growth, and physical activity at baseline. In addition, these differences were perhaps due to the changing COVID-19 measures. The current study was conducted at the beginning of the second wave of the COVID-19 outbreak in Hong Kong, while our previous RCT (21) was conducted at the later stage of the same outbreak. The tightened social distancing measures, such as prohibited group gatherings and dine-in ban, and lockdown protocols implemented during the early stage of the outbreak might have had a more significant impact on individuals’ daily functioning (e.g., work, school, social), activity level, and quality of life, given they had less time to process and adapt to the changes (55, 56). It is possible that a stronger result would have resulted in the absence of COVID-19 restrictions. Further studies are warranted to fully understand the efficacy of Lifestyle Hub for improving mental health in nonclinical populations.

In our study, the LH group demonstrated a significantly higher (20.8%) study attrition rate than the WL control group (5.7%). Despite a higher attrition rate relative to our previous RCT (21), the LH group in the current study reported a lower attrition rate than general smartphone-delivered interventions for improving mental health in nonclinical populations (i.e., 27%) (57). We were unable to determine specific reasons for study attrition in the LH group because most of the participants who dropped out were not contactable at post-intervention time points. The significant difference in study attrition between the LH and WL groups may be attributable to our study design. A previous meta-analysis suggested that participants in WL are more motivated to remain in the trial as compared to participants in the intervention group who already had access to all the intervention content (57). Another possibility may be related to the fact that Lifestyle Hub was a pure self-help intervention without any human encouragement or support provided to the participants (58).

While this RCT has contributed to the body of evidence supporting the efficacy of the LM approach in improving mental health in nonclinical populations, the results should be considered in light of the following potential limitations. Despite utilizing open recruitment strategies to enhance the generalizability of the sample, the included sample was predominantly female (77.4%) and those with higher educational attainment (75.5%). Moreover, the lack of blinding of participants might threaten the internal validity of the study findings. In addition, the potential improvements in outcomes may be masked by the floor effect, given a nonclinical sample was targeted in this RCT. Besides, the medium- and long-term effects of Lifestyle Hub are unclear, considering that the only follow-up assessment was conducted at 1-month post-intervention. Further investigation into the durability of the Lifestyle Hub is needed since the LM approach stresses long-term benefits (15).

This RCT represents a pioneer attempt to investigate the efficacy of a smartphone-delivered multicomponent LM intervention for improving mental health among a nonclinical population. Several research endeavors are important to be considered in future literature. First, future trials that include participants with a variety of diagnostic profiles (e.g., depression, anxiety, and /or insomnia) are warranted to establish the transdiagnostic potential of the LM approach. Additionally, it is crucial for upcoming causal research to understand the direct impacts of LM on CMDs and the underlying mechanisms of change. Concurrently, identifying the potential moderators and mediators as well as delineating the direct and indirect effects of LM on CMDs are also important (17). Second, therapy outcome studies that employ the dismantling design are needed to identify active intervention components that drive the observed clinical effects (59). Such investigation could lead to the optimization and inform the development of more streamlined and cost-effective intervention protocols. Third, future studies could enhance the depth of their findings by utilizing qualitative research methods (e.g., focus groups, interviews) to gain insight into the processes by which participants initiate and sustain lifestyle modifications. Also, qualitative investigations could be employed to elucidate specific intervention components and features that yield favorable experiences for participants as well as to evaluate intervention satisfaction. Fourth, future research might enroll participants with more severe depressive and anxiety symptomatology to provide more robust evidence for the clinical utility of smartphone-delivered lifestyle medicine interventions (14, 16, 17, 21). Lastly, while this RCT has provided support for greater dissemination and accessibility of smartphone-delivered lifestyle-based mental health care, it remains unclear how these interventions can be effectively integrated into the current mental health systems and delivered at the population level. As recommended by recent guidelines, a potential option may be incorporating lifestyle-based mental health care as the initial step within a stepped care model for CMDs (11, 22–24). Future clinical trials and cost-effectiveness analyses are warranted to investigate this possibility.

In summary, smartphone-delivered multicomponent LM intervention may serve as an efficacious, safe, and acceptable option for improving overall mental health conditions, insomnia severity, overall HPBs, dietary quality, and stress management in nonclinical adult populations. Future research is needed to investigate the long-term efficacy of Lifestyle Hub and how to maximize the benefits of smartphone-delivered LM interventions at the population level.

## Data availability statement

The datasets presented in this article are not readily available because the data underlying this article will be shared on reasonable request to the corresponding author. Requests to access the datasets should be directed to FY-YH, fionahoyy@cuhk.edu.hk.

## Ethics statement

The studies involving humans were approved by Survey and Behavioral Research Ethics Committee, The Chinese University of Hong Kong. The studies were conducted in accordance with the local legislation and institutional requirements. The participants provided their written informed consent to participate in this study.

## Author contributions

VWW and FY-YH designed the study protocol, developed the intervention, performed the data analysis, and contributed to the writing of the manuscript. JTT and N-KS assisted in developing the intervention and data collection and contributed to the writing of the manuscript. CN and JS verified the intervention content and contributed to the writing and reviewing of the manuscript. All authors contributed to the article and approved the submitted version.
