# Corrigendum: Smartphone-delivered multicomponent lifestyle medicine intervention for improving mental health in a nonclinical population: a randomized controlled trial

**DOI:** 10.3389/fpubh.2024.1389847

**Published:** 2024-03-04

**Authors:** Vincent Wing-Hei Wong, Jessica Tsz-Yan Tong, Nga-Kwan Shi, Chee H. Ng, Jerome Sarris, Fiona Yan-Yee Ho

**Affiliations:** ^1^Department of Psychology, The Chinese University of Hong Kong, Shatin, Hong Kong, China; ^2^Department of Psychiatry, The Melbourne Clinic and St Vincent's Hospital, University of Melbourne, Richmond, VIC, Australia; ^3^Department of Psychiatry, Professorial Unit, The Melbourne Clinic, The University of Melbourne, Melbourne, VIC, Australia; ^4^Western Sydney University, NICM Health Research Institute, Westmead, NSW, Australia

**Keywords:** lifestyle, mood, mental health, self-help, smartphone-based intervention, randomized controlled trial

In the published article, there was an error in the Conflict of Interest statement. The original Conflict of Interest statement was as follows: “The authors declare that the research was conducted in the absence of any commercial or financial relationships that could be construed as a potential conflict of interest”. The correct Conflict of Interest statement appears below.

